# Robot-assisted conversion of ureterocutaneous stoma to ileal conduit: a novel option for patients with post-cystectomy cutaneous diversion complicated by ureteral stricture and recurrent infections

**DOI:** 10.3389/fruro.2025.1634602

**Published:** 2025-08-15

**Authors:** Tengfei Gu, Ting Chen, Yongtao Pan, Qinzhou Yu, Jie Li

**Affiliations:** Department of Urology, Lishui Municipal Central Hospital, The Fifth Affiliated Hospital of Wenzhou Medical University, Lishui, China

**Keywords:** robot-assisted, ureterocutaneostomy, ileal channel stoma, urinary diversion, total cystectomy

## Abstract

**Background:**

Radical cystectomy accompanied by urinary diversion remains the standard surgical intervention for individuals diagnosed with muscle-invasive bladder cancer. Notably, around 30% of these patients opt for a ureterocutaneous stoma. However, this technique is frequently associated with complications such as anastomotic stenosis, obstruction, and infection, which can lead to the deterioration of renal function and significantly impair the patient’s quality of life. Consequently, this study investigates the safety of robot-assisted laparoscopic conversion from a ureterocutaneous stoma to an ileal conduit stoma, thereby offering a novel surgical alternative for patients undergoing total cystectomy with ureterocutaneous stoma.

**Methods:**

A retrospective analysis was carried out on two patients who underwent total cystectomy and ureterocutaneous stoma and were admitted to our hospital in January 2025. We performed robot-assisted laparoscopic conversion of the ureterocutaneous stoma to an ileal conduit for these patients and subsequently evaluated the clinical benefits and surgical safety associated with the procedure.

**Result:**

Both patients successfully underwent surgery, with operation durations of 293 minutes and 281 minutes, respectively. Intraoperative blood loss was recorded at 100 ml and 50 ml, respectively. Abdominal drainage tubes were removed five days postoperatively, and both patients were discharged seven days following the procedure. No surgery-related complications were observed during the perioperative period. Ureteral stents were removed two months post-surgery. Post-extubation CT scans indicated a resolution of the initial mild hydronephrosis in the kidneys. Renal function assessments, including creatinine levels and glomerular filtration rate, demonstrated improvement compared to preoperative values. Additionally, patients reported lower pain scores and higher quality of life scores postoperatively compared to preoperative assessments.

**Conclusion:**

Robot-assisted laparoscopic ureterocutaneostomy to ileal channel surgery is both feasible and safe, offering potential improvements in renal function and quality of life for patients. Additionally, it presents an alternative surgical option for those requiring ureterocutaneostomy.

## 1 Introduction

Radical cystectomy is the standard surgical intervention for the management of muscle-invasive bladder cancer or complex and refractory non-muscle-invasive bladder cancer (1). Urinary diversion, a critical component of this procedure, significantly influences both the postoperative quality of life and the long-term prognosis of patients. Currently, the primary urinary diversion techniques employed in clinical practice include ileal conduit surgery (Bricker procedure), orthotopic neobladder reconstruction, and ureterocutaneostomy (2). Ureterocutaneostomy is frequently utilized in elderly patients or those with significant comorbidities due to its relative procedural simplicity and reduced operative duration (3). However, this approach is associated with a relatively high incidence of long-term complications, such as stoma stenosis (8%-15%), urinary tract infections (20%-30%), parastomal hernia (5%-10%), and skin irritation dermatitis (25%-40%), all of which can substantially impact patient quality of life (4). Consequently, some patients may require subsequent surgical interventions for complication management.

A percutaneous nephrostomy or indwelling ureteral stents was usually used to relieve hydronephrosis caused by ureterocutaneostomy stenosis. However, percutaneous nephrostomy or the insertion of ureteral stents could not only impair the quality of life but also lead to infection, stones, and even deterioration of renal function (5).For patients necessitating urinary diversion repair, traditional open surgery, while capable of reconstructing the ileal channel, encounters several challenges, including the difficulty of separating abdominal adhesions, limited ureteral length, and stringent requirements for anastomosis precision. The incidence of postoperative complications, such as intestinal obstruction and urinary leakage, ranges from 12% to 25% (6). Although laparoscopic techniques offer the benefits of minimally invasive surgery, they are constrained by limited operational freedom, particularly in executing precise ureterointestinal anastomosis under a complete endoscopic view. In recent years, robot-assisted laparoscopic surgery systems have markedly enhanced the accuracy and safety of complex urinary tract reconstruction procedures (7). This advancement is attributed to their three-dimensional high-definition visualization, the 7-degree-of-freedom movement of EndoWrist bionic instruments, and tremor-filtering capabilities (8). Research indicates that the incidence of stenosis in ureterointestinal anastomosis performed via robotic surgery can be reduced to 3%-5% (9).Furthermore, robotic surgery minimizes the risk of intraoperative bleeding and damage to surrounding organs through more precise tissue dissection (10).

In view of the emerging application of robotic repair surgery in complex urinary tract reconstruction at present, this article aims to preliminarily explore the feasibility and operational key points of robot-assisted ureterocutaneostomy to ileal conduit stoma conversion through two technical reports, providing technical references for clinical practice.

## 2 Materials and methods

### 2.1 Patient information

The study was approved by the Ethics Committee of Lishui Central Hospital in Zhejiang, China, using a retrospective design. In January 2025, Retrospective collection two patients who had undergone total cystectomy presented with ureterocutaneostomy and subsequently underwent robot-assisted laparoscopic ureterocutaneostomy to ileal conduit anastomosis. A retrospective analysis of the pertinent clinical data was conducted to evaluate the efficacy and safety of this surgical technique.

Patients involves a 49-year-old female patient who underwent a total cystectomy with ureterostomy on January 2, 2024, due to a malignant bladder tumor. Histopathological examination revealed high-grade invasive urothelial carcinoma with myometrial infiltration. The postoperative staging was determined to be T2N0M0. A ureteral stent was placed postoperatively and has been replaced at three-month intervals. Within the first year following surgery, the patient required hospitalization on two occasions due to infection and fever. Additionally, there is evidence of mild renal function impairment. Another patient involves a 64-year-old male patient who underwent transurethral resection of a bladder tumor on December 23, 2022, due to the presence of a bladder tumor. Postoperative pathological analysis revealed high-grade invasive urothelial carcinoma infiltrating the muscular layer, with a clinical stage of T2N0M0. Subsequently, on January 5, 2023, the patient underwent a total cystectomy combined with ureterostomy. Postoperative pathology indicated that the tumor had invaded the outer membrane of the bladder, resulting in an updated clinical stage of T3N0M0. The patient received postoperative adjuvant therapy consisting of vedisitumab combined with pembrolizumab. Following the surgery, the ureteral stent was replaced regularly. Over the course of two years, the patient was hospitalized six times for treatment due to infection and fever, and experienced mild renal function impairment. Both patients presented with severe hydronephrosis of the left kidney. Both patients underwent transperitoneal ureterocutaneostomy (UCS) during their radical cystectomy, with the ureters positioned intraperitoneally.

Initially, both patients opted for ureterocutaneostomy as their urinary diversion method. However, prolonged indwelling of ureteral stents led to recurrent urinary tract infections, compromised renal function, and other complications, significantly diminishing their quality of life. Consequently, the patients expressed a strong desire to modify their urinary diversion approach. In response, we conducted a robot-assisted laparoscopic procedure to convert the ureterocutaneostomy to an ileal conduit stoma for both individuals.

### 2.2 Surgical process

**Figure 1:**
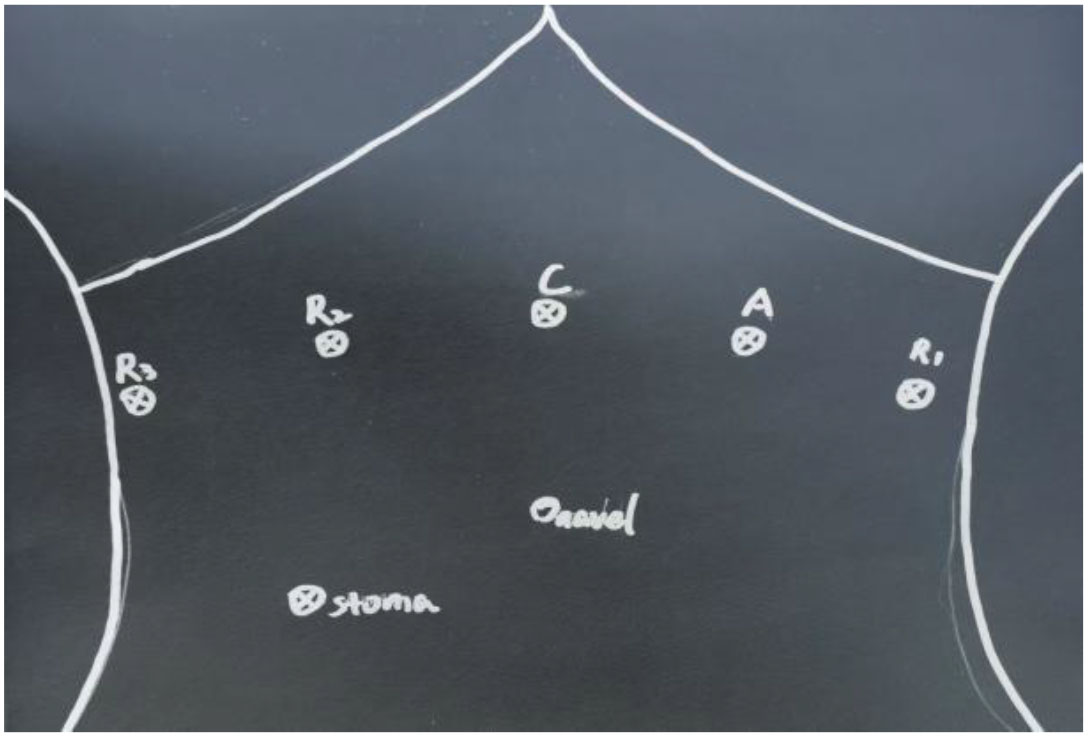
Layout of puncture holes and cannulas.

**Figure 2:**
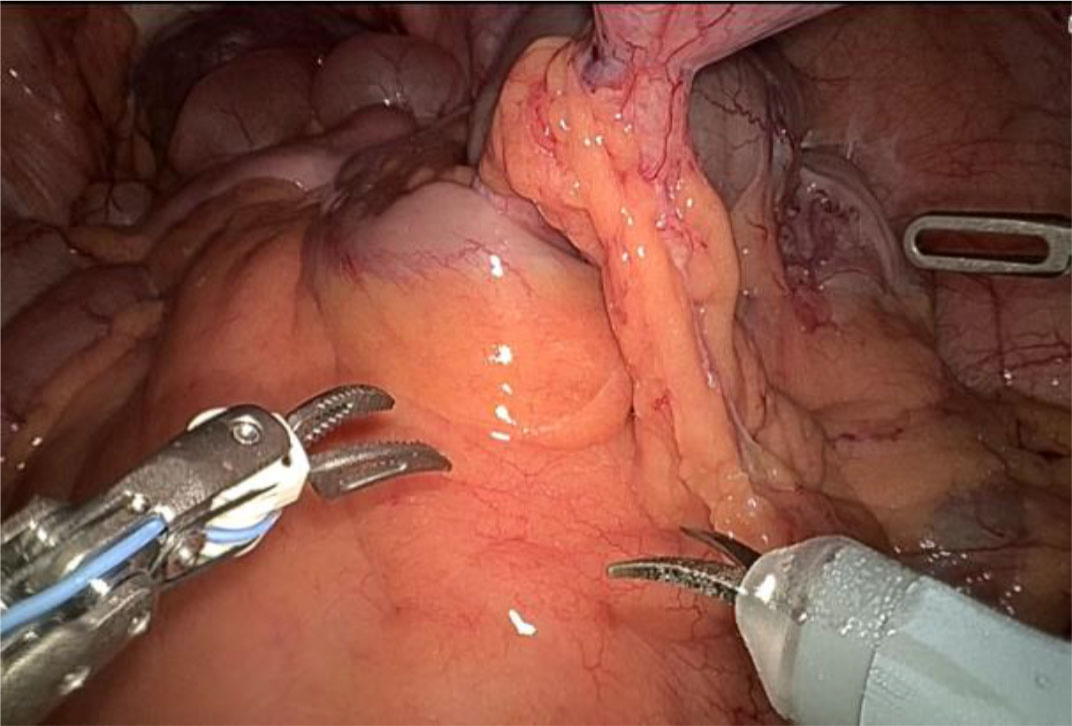
Exploration of abdominal adhesion.

**Figure 3:**
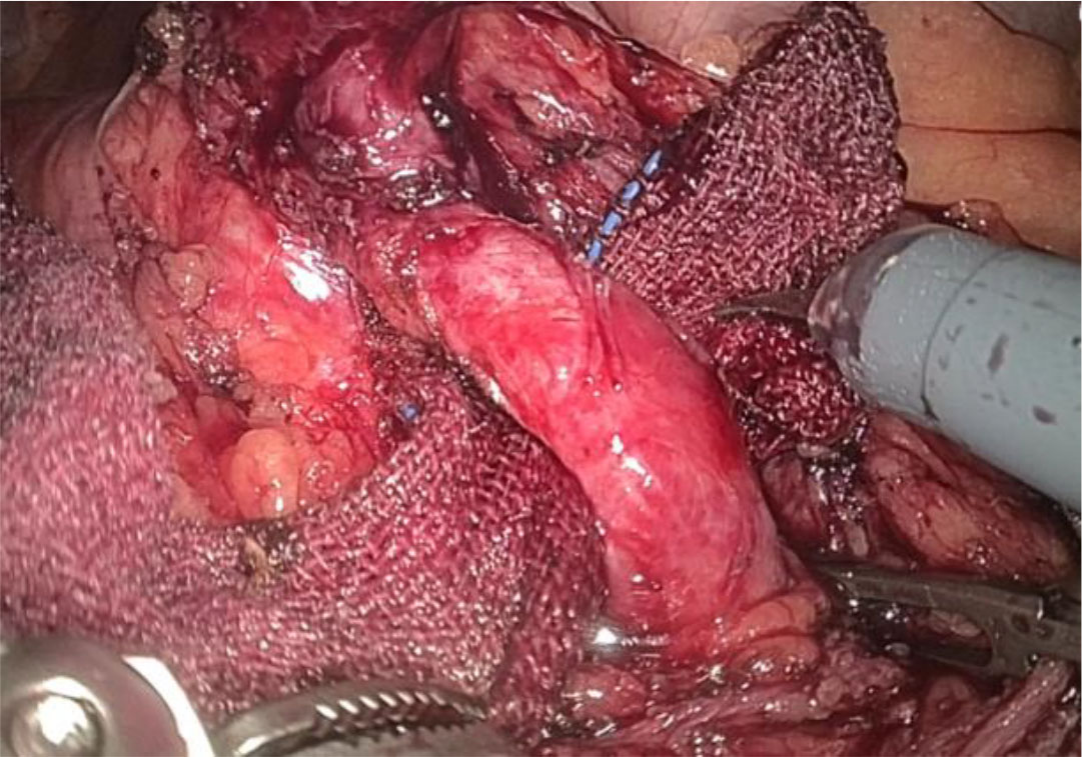
Separates the right ureter.

**Figure 4:**
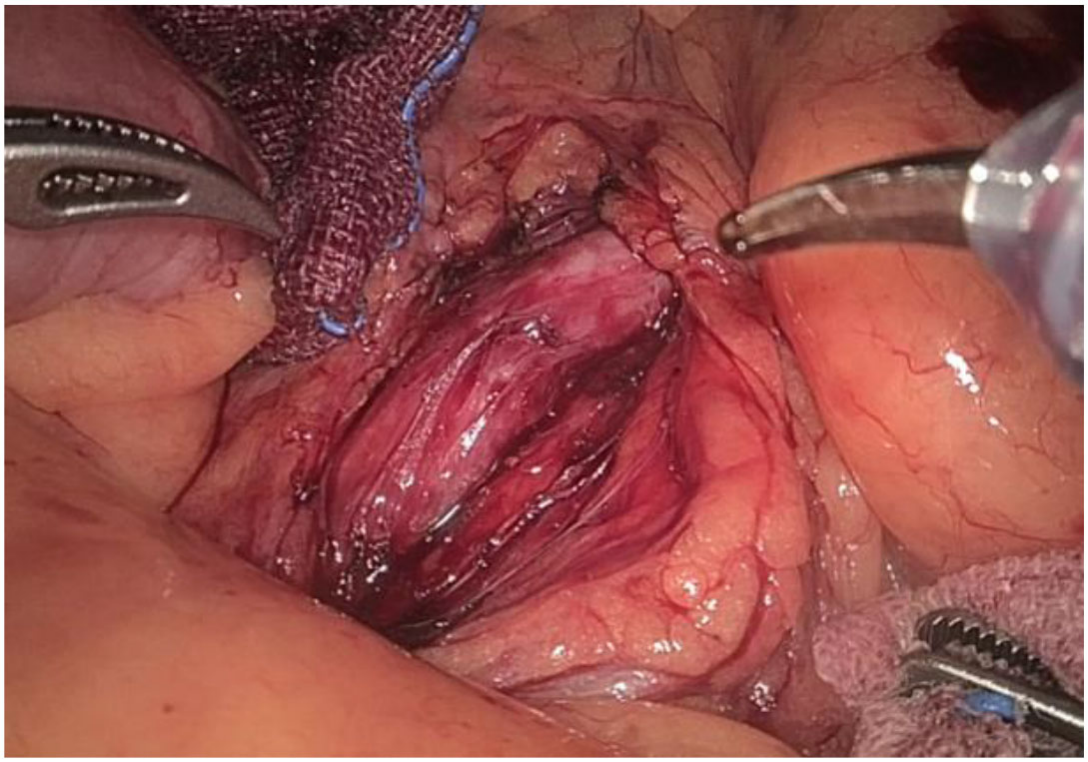
Separates the left ureter.

**Figure 5:**
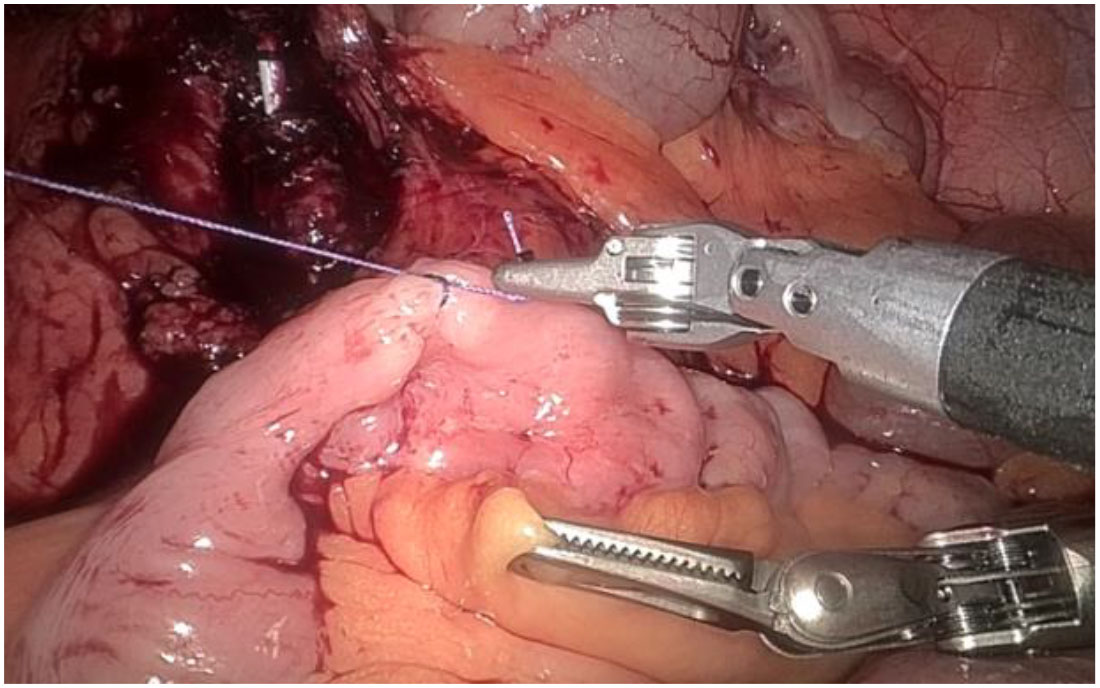
Marks the selected ileum.

**Figure 6:**
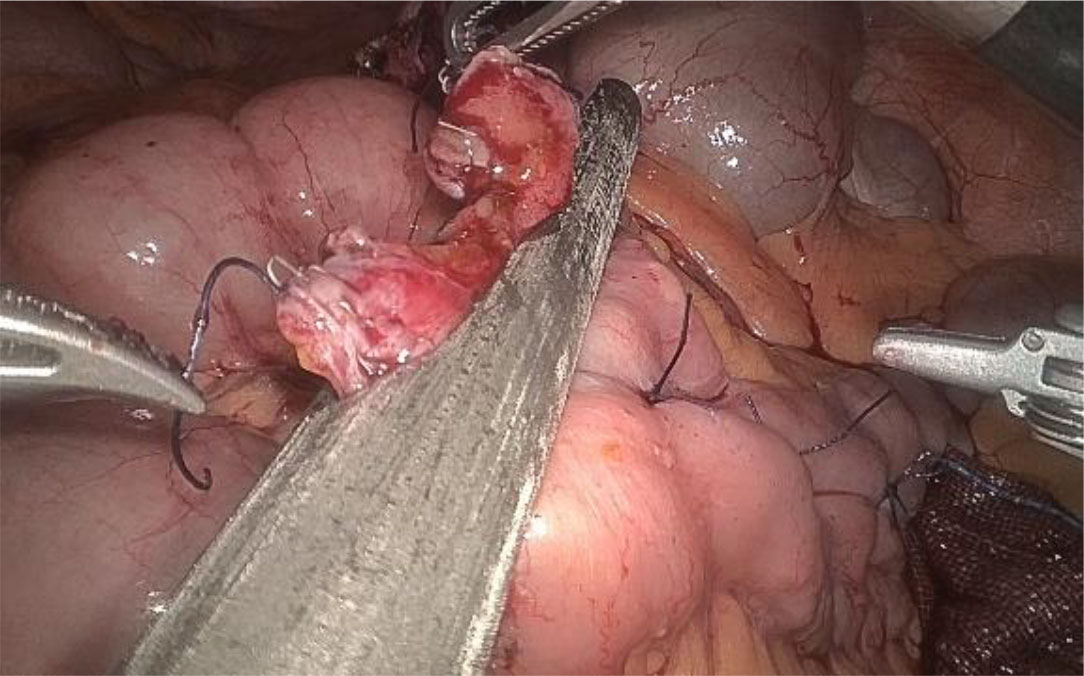
Restoring the continuity of the intestinal tract.

**Figure 7:**
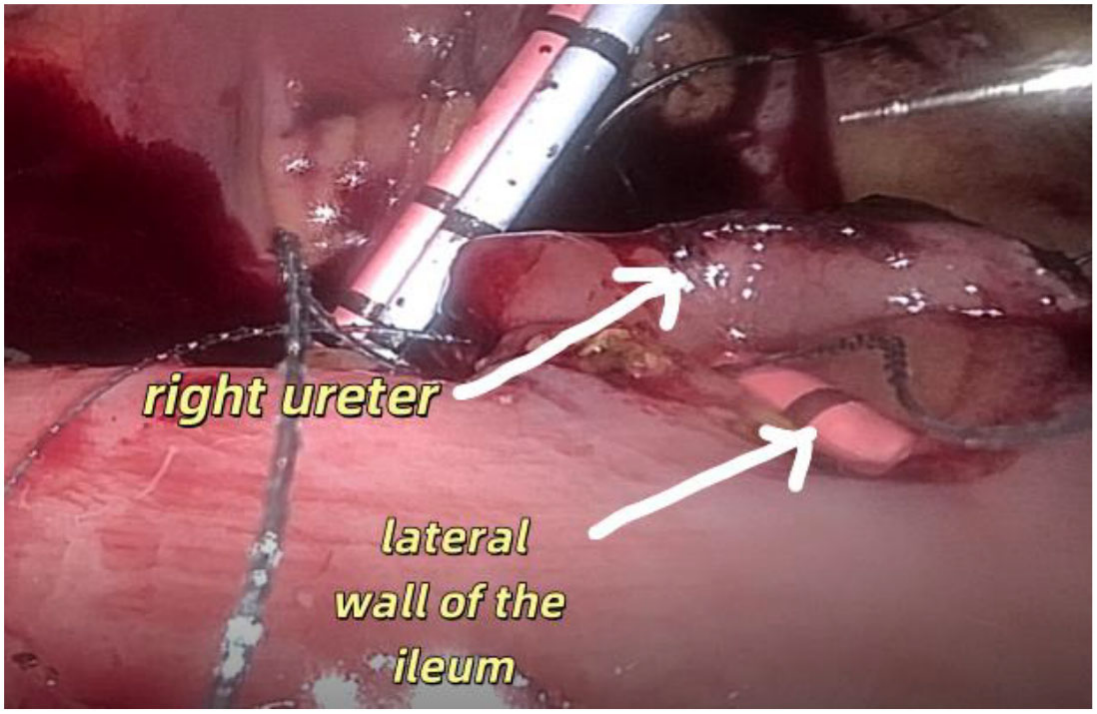
Shows that the right ureter is anastomosed with.

**Figure 8:**
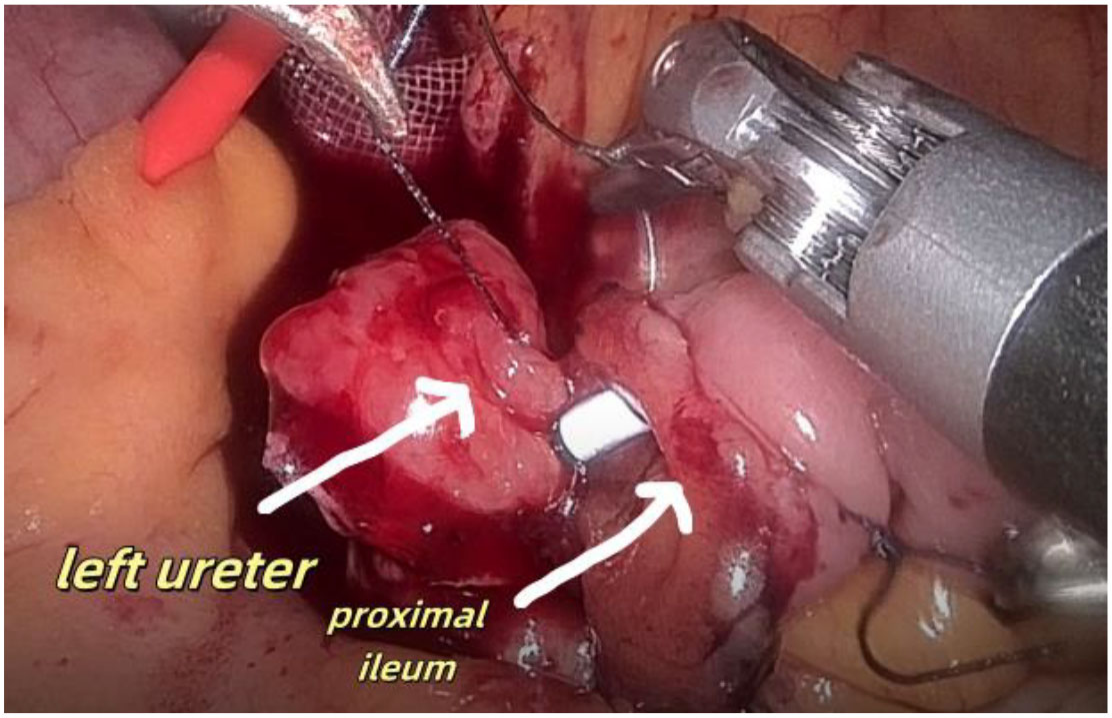
Shows anastomosis between the left ureter and the lateral wall of the ileum the proximal ileum.

**Figure 9:**
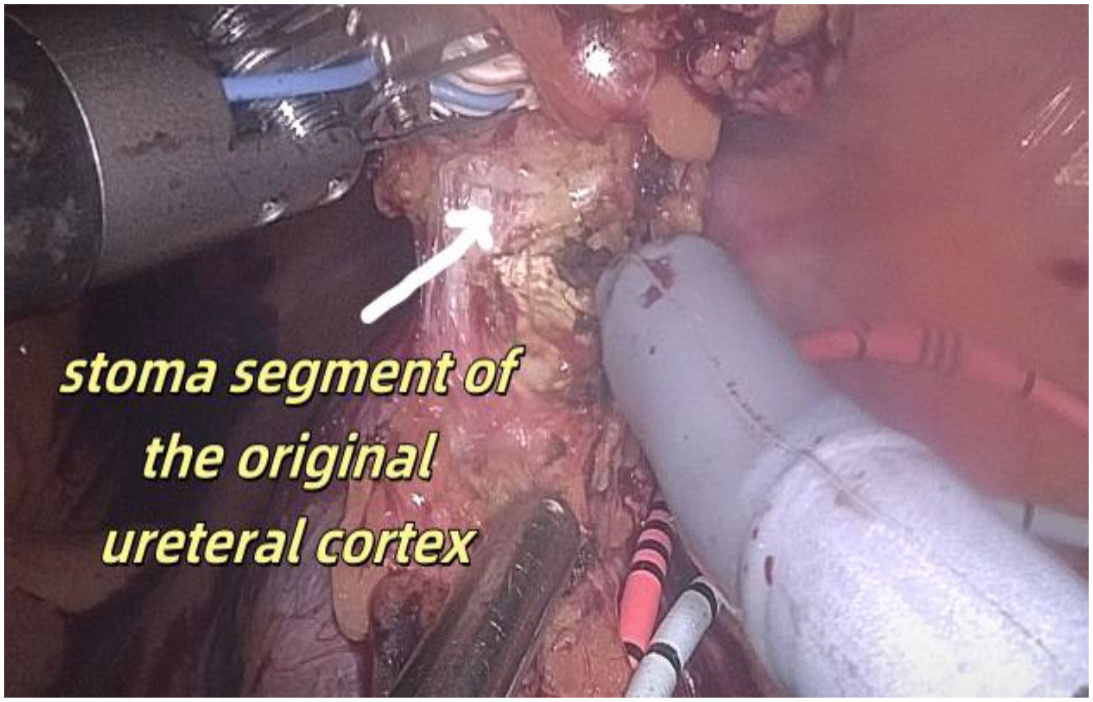
Resection of the stoma segment of the original ureteral cortex.

**Figure 10:**
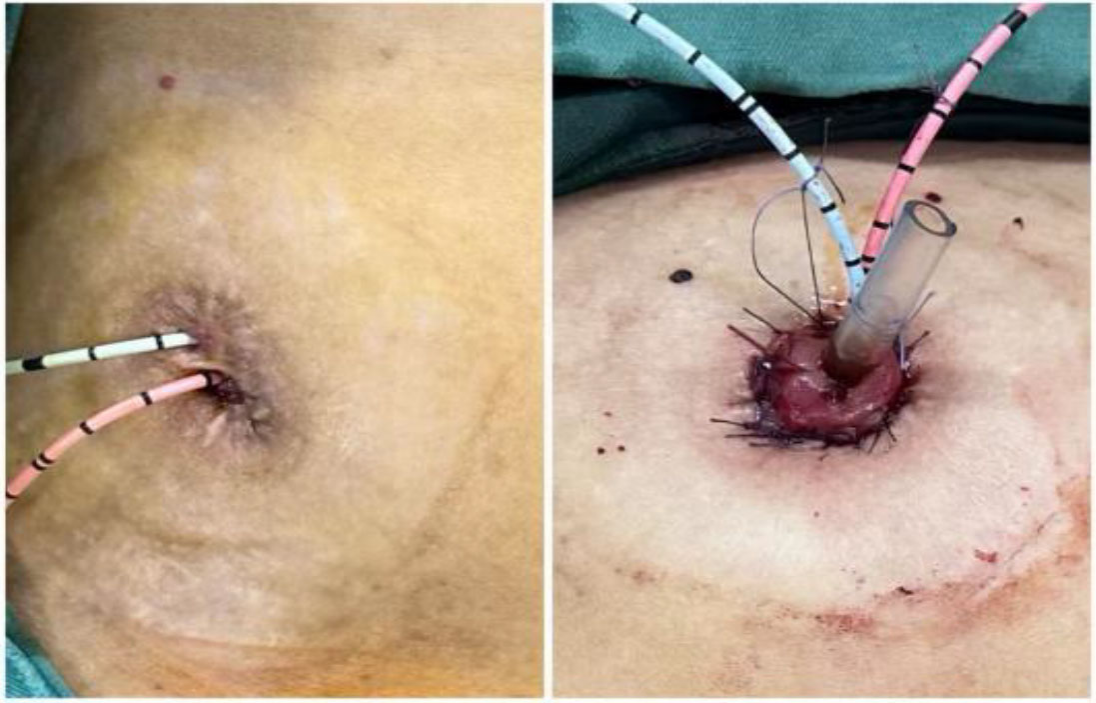
Re-ileostomy.

1. Installation: The patient was positioned supine, and the robotic sleeve was placed and punctured to establish pneumoperitoneum. Subsequently, the robotic arm was installed to facilitate exploration of abdominal cavity adhesions (refer to **Figures 1**, **2**). 2. Ureteral Separation: The right ureter was isolated and transected at the iliac vessel location to prepare for anastomosis. The left ureter was identified at the left mesenteric hiatus and similarly transected for anastomosis (refer to **Figures 3**, **4**). 3. Selection of the Intestinal Segment: A segment of the ileum, approximately 15 cm proximal to the ileocecal junction, and then restore the continuity of the ileum. 4. Ureteroileal Anastomosis: The left ureter was anastomosed to the proximal end of the ileum, while the right ureter was connected to the lateral wall of the ileum. A ureteral stent was placed under direct visualization to ensure unobstructed urine flow. 5. Ileostomy: The original ureteral cutaneous stoma in the right lower abdomen was excised, and the channel was expanded. The peritoneum and anterior sheath of the rectus abdominis were sutured and secured at the incision site. The ileal segment was exteriorized, with care taken to preserve mesenteric blood supply, and everted to form a papilla. A drainage tube was inserted to facilitate postoperative management (**Figures 1**–**10**).

## 3 Result

### 3.1 Comparison of changes in hydronephrosis in patients

The hydronephrosis of the patients after the operation was significantly improved compared with that before the operation.

### 3.2 Comparison of changes in patients’ renal function

The postoperative serum creatinine and glomerular filtration rate of both patients were improved compared with those before the operation.

The postoperative serum creatinine and glomerular filtration rate of both patients were improved compared with those before the operation.

### 3.3 Relevant indicators of the patient during the perioperative period

The surgical procedures of the two patients went smoothly. No secondary injuries to other tissues or organs occurred during the operation. No serious complications occurred after the operation, and the tubes were removed and the patients were discharged smoothly after the operation.

### 3.4 Comparison of the time of ureteral stent removal, pain score(visual simulation scale, VAS) and quality of life score(QoL scale) after extubation in patients

Both patients have been followed up for 5 months postoperatively after undergoing robotic intracorporeal ileal conduit (IC) diversion. They successfully removed the ureteral stents about 2 months after the operation. The pain score and quality of life score after extubation were significantly improved compared with those before the operation.

## 4 Discussion

Radical cystectomy remains the standard surgical approach for managing muscle-invasive bladder cancer. The ureterocutaneous stoma is frequently employed in clinical settings due to its straightforward procedure and reduced operative duration. Nonetheless, it is associated with a relatively high incidence of long-term complications, which significantly impact renal function and the overall quality of life for patients (11). Compared with ileal conduit stoma, ureterocutaneous stoma has a higher incidence of stenosis, obstruction and infection (12–14). Since the blood supply is better after anastomosis between the ileal mucosa and ureteral mucosa, the incidence of stenosis is lower. Additionally, peristalsis of the ileum helps reduce urinary stasis, thereby decreasing the risks of obstruction and infection. However, perioperative safety needs to be taken into consideration. Consequently, there is an urgent need to investigate alternative surgical techniques that offer new options for patients requiring a change in urinary diversion from a ureterocutaneous stoma. This study conducts a retrospective analysis of two cases involving the conversion from a ureterocutaneous stoma to an ileal conduit, providing preliminary evidence supporting the feasibility and safety of this surgical approach. The technical advantages and clinical significance of this method merit further comprehensive investigation.

Based on our observations, the left ureter carries a higher risk of stomal stenosis following ureterocutaneostomy. The significantly higher incidence of strictures and functional impairment in the left ureter following ureterocutaneostomyis attributable to its unique anatomical challenges during mobilization. To reach the right-sided abdominal stoma, the left ureter requires extensive dissection, crossing the midline beneath the sigmoid mesentery and aorta. This elongated course increases susceptibility to: vascular compromise, tension and angulation and retroperitoneal tunnel issues (15).

The revision surgery for urinary diversion following total cystectomy presents significant challenges. The presence of extensive adhesions involving the intestinal tract and ureter within the abdominal cavity postoperatively introduces a level of uncertainty, contributing to a higher incidence of complications associated with traditional open or laparoscopic surgical approaches (16). This complexity often restricts the application of these surgical methods. However, the advent of robot-assisted laparoscopic technology has markedly enhanced the precision of complex urinary tract reconstruction procedures (17). In this study, both patients successfully underwent uretero-ileal anastomosis without experiencing complications such as anastomotic stenosis or urinary leakage postoperatively. The direct anastomosis between the ileal mucosa and the ureter is advantageous in minimizing scar formation. According to the literature, the rate of anastomotic stenosis with this approach (3%-5%) is significantly lower compared to that associated with ureterocutaneous stoma (3). In comparison to traditional open surgery, which necessitates extensive dissection of abdominal adhesions and is challenged by limited ureteral length and low anastomotic precision, the robotic system offers the capability to meticulously release the ureter through a minimal incision and perform complex anastomoses within confined spaces, thereby minimizing excessive traction on surrounding tissues (18). Furthermore, robotic surgery provides enhanced flexibility in the selection and anastomosis of the ileal segment. The robotic system can simulate human wrist movements, allowing precise dissection of mesenteric vessels within the confined pelvic space. Its 3D high-definition visualization clearly identifies the mesenteric vascular arcades, preventing inadvertent injury and ensuring optimal blood supply to the selected ileal segment. The dexterity of robotic instruments enables surgeons to directly measure the required ileal segment intraoperatively. Real-time assessment allows for adjustments in resection length, avoiding excessive tension from insufficient segment length or urinary stasis caused by redundant loops. These technical advantages may contribute to reduced operative time and improved outcomes in intracorporeal urinary diversion procedures. In this study, the postoperative intestinal function of both patients demonstrated favorable recovery, with no incidence of intestinal obstruction, indicating that robotic surgery may effectively mitigate the risk of complications associated with intestinal procedures in conventional surgical approaches.

**Figure 11:**
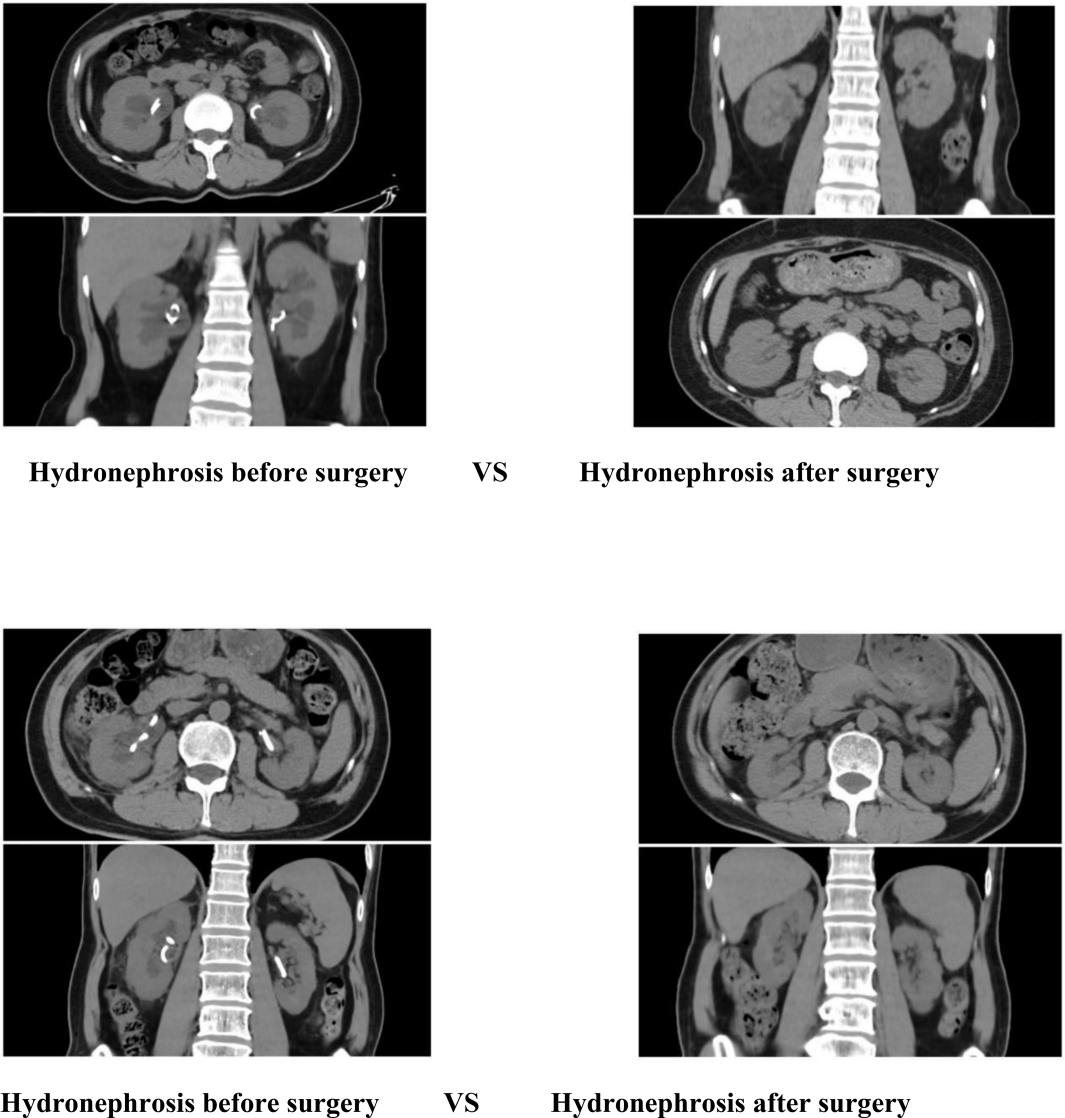
Hydronephrosis before surgery VS Hydronephrosis after surgery.

**Figure 12:**
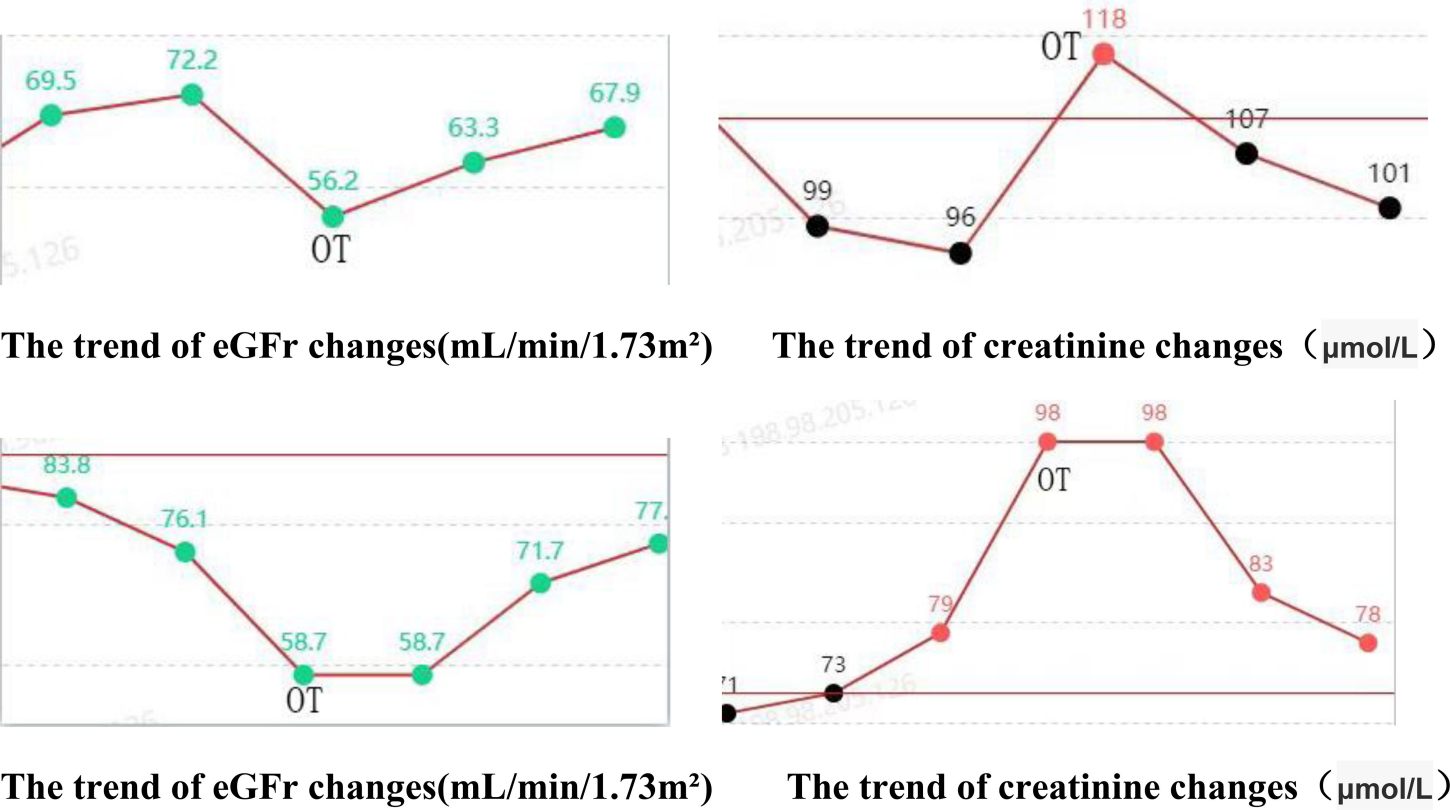
Hydronephrosis before surgery VS Hydronephrosis after surgery.

The findings of this study indicate that the robotic surgery had operation times of 293 minutes and 281 minutes, respectively (**Table 1**). Blood loss was minimal, ranging from approximately 50 to 100 ml. Postoperative outcomes were favorable, with no significant complications such as infection, hemorrhage, or intestinal obstruction observed. The drainage tube was removed five days post-surgery, and the patient was discharged on the seventh day, aligning with the principles of enhanced recovery after surgery. Although the duration of the operation was relatively extended, it was comparable to the average duration of 3 to 4 hours for open repair surgeries documented in the literature (19). The time required for the precise maneuvers of the robotic system in complex tasks such as adhesion separation, ureteral dissection, and anastomosis was deemed appropriate. Furthermore, in comparison to the reported complication rates of 12% to 25% for traditional open repair surgeries, the risk of complications associated with robotic surgery was significantly lower (20). This reduction in risk can likely be attributed to the robotic system’s precision, which minimizes tissue trauma and intraoperative bleeding. Furthermore, stenosis of the ureteral cutaneous stoma or obstruction related to stents frequently leads to hydronephrosis, which subsequently results in a reduction in the estimated glomerular filtration rate (eGFR) and an elevation in serum creatinine levels, thereby accelerating the progression of chronic kidney disease (21). In this cohort, all patients exhibited mild hydronephrosis and impaired renal function preoperatively. Postoperatively, there was an improvement in renal function, evidenced by a decrease in creatinine levels, an increase in eGFR, and a regression of hydronephrosis (**Figures 11**, **12**). These findings further substantiate the efficacy of this surgical approach in alleviating urinary tract obstruction, thereby decelerating the deterioration of renal function. This improvement may be attributed to the peristaltic activity of the ileal conduit, which facilitates urine drainage, reduces the risk of residual urine and reflux, and consequently mitigates hydronephrosis while preserving renal function (22).

**Table 1:** Relevant indicators of the patient during the perioperative period.

Indicator	Operation time (min)	Operation bleeding(ml)	Intestinal injury (N/Y)	Postoperative bleeding (N/Y)	Preoperative hemoglobin (g/L)	Postoperative hemoglobin (g/L)	Fever(>38.5°C) (N/Y)	Intestinal obstruction (N/Y)	Time of drainage tube (day)	Hospital stay (day)
Case1	293	100	N	N	123	111	N	N	5	7
Case2	281	50	N	N	118	108	N	N	5	7

Patients with a ureterocutaneous stoma are required to have ureteral stents indwelling for extended periods, necessitating frequent stent replacements, managing peristomal skin complications, and enduring the psychological and economic burdens associated with recurrent hospitalizations (23). Additionally, the prolonged presence of stent tubes increases the risk of infections, which can lead to varying degrees of pain and significantly impair patients’ quality of life (24). In contrast, ileostomy does not necessitate the long-term indwelling of ureteral stents. In the two patients studied, the ureteral stents were removed 58 and 62 days postoperatively, aligning with the standard management protocol of 6–8 weeks following ileostomy. Furthermore, stoma care has become more convenient, leading to significant improvements in both pain and quality of life scores for patients. Specifically, the Visual Analogue Scale (VAS) scores for two patients decreased from 4 and 5 points preoperatively to 0 and 1 point, respectively, following extubation. This alleviation of pain is attributed to the prevention of peri-stoma dermatitis of the ureteral skin and the reduction of long-term irritation caused by indwelling ureteral stents post-ileostomy. Literature indicates that patients undergoing total cystectomy with ileostomy experience a higher quality of life compared to those with ureterocutaneous stomy (25) (**Table 2**).

**Table 2:** Comparison of the time of ureteral stent removal, pain score (Visual Simulation Scale, VAS) and quality of life score (QoL Scale) after extubation in patients.

Indicator	Time of ureteral stent removal (day)	Preoperative pain score (VAS)	Postoperative pain score (VAS)	Preoperative quality of life score	Postoperative quality of life score
Case1	58	4	0	5	8
Case2	62	5	1	4	8

For patients experiencing postoperative complications associated with a ureterocutaneous stoma, transitioning to an ileal channel stoma represents not only a critical intervention for alleviating obstruction and preserving renal function but also an essential strategy for enhancing patients’ social engagement and psychological resilience. The advent of robot-assisted laparoscopic technology has significantly surpassed the constraints of conventional open surgery. Although there have been reports of robotic repair of ureterointestinal anastomotic stenosis, this study systematically describe the complete robotic operation process of UCS to IC conversion, and confirm its unique value in protecting renal function.In this study, both patients opted for reoperation due to recurrent infections and compromised renal function following ureterocutaneous stoma. Although their clinical profiles were illustrative, the study was limited by a small sample size and a lack of long-term follow-up data, but neither of the two patients had anastomotic leakage or stenosis after the operation, and the QoL score improved by ≥40%, which provided a basis for subsequent prospective studies. Additionally, the study’s design as a single-center retrospective analysis without a control group precluded direct comparisons between robotic and traditional surgical approaches. Future research should aim to increase the sample size, extend the duration of follow-up, and validate the findings through multicenter randomized controlled trials.

In patients experiencing ureterocutaneous stoma complications following total cystectomy, robot-assisted laparoscopic repair surgery offers a minimally invasive and precise therapeutic approach, potentially enhancing long-term patient outcomes. As robotic technology becomes more widespread and surgeons gain greater experience, the associated learning curve is likely to decrease, facilitating the broader adoption of this surgical technique. Future advancements, including artificial intelligence-assisted planning and intraoperative navigation, warrant further investigation to optimize surgical procedures and enhance therapeutic efficacy.

In conclusion, this study provides preliminary confirmation of the feasibility and safety of robot-assisted laparoscopic ureterocutaneous stoma to ileal channel stoma surgery. The technical advantages demonstrated in this procedure hold substantial value for complex urinary tract reconstruction. Although the study is limited by a small sample size, the findings establish a robust foundation for future research and introduce novel concepts for clinical practice.

## Data Availability

The original contributions presented in the study are included in the article/supplementary material. Further inquiries can be directed to the corresponding author.
